# A rare case report of osteochondroma of the left medial cuneiform

**DOI:** 10.1097/MS9.0000000000002358

**Published:** 2024-07-08

**Authors:** Rohit Shrestha, Sandesh S. Maharjan, Abhishek Pandey, Kriti Pradhananga, Archana Pandey

**Affiliations:** a Kathmandu University School of Medical Sciences, Dhulikhel Hospital; b Department of Orthopedics and Traumatology, Dhulikhel Hospital, Dhulikhel, Nepal

**Keywords:** case report, cuneiform, osteochondroma

## Abstract

**Introduction and importance:**

Osteochondroma is a benign skeletal neoplasm presenting with the proliferation of bony tissue. Osteochondroma of the foot is uncommon, and that of the cuneiform is an extremely rare entity.

**Case presentation:**

We present the case of a 22-year-old woman with osteochondroma of the left medial cuneiform who was having pain in the first ray of the left foot.

**Clinical discussion:**

Most cases of osteochondromas are nontender and painless masses with a benign asymptomatic course; however, progression to inflammation and neurovascular complications may cause considerable morbidity. The majority of cases are treated conservatively, while some severe cases require surgical management.

**Conclusion:**

Operative treatment of osteochondroma with excision of the mass remains a safe and successful alternative to conservative management whenever required.

## Introduction

Highlights

- Osteochondroma is a common benign tumor characterized by the abnormal proliferation of bony tissue with a cartilaginous cap.
- They are usually asymptomatic and found incidentally, but they can cause pain and discomfort if complicated by inflammation, fracture, nerve compression, or malignancy.
- Osteochondromas usually occur in the metaphysis of long bones, but this case has one in the rare location of the cuneiform tarsal bone.
- Surgical excision of osteochondroma is a valuable alternative to conservative management when symptoms persist.

Osteochondroma is the most common benign skeletal tumor, accounting for about 50% of benign skeletal neoplasms and 15% of all bony neoplasms^1^. It presents as an abnormal proliferation of bony tissue with a cartilaginous cap around areas of active bone growth. Osteochondromas are most commonly solitary (90%) but can be a part of multiple hereditary exostosis^2–4^. They are generally asymptomatic and detected incidentally on radiographs as pedunculated or sessile osseous protuberance from the bone surface. They may, however, present with symptoms like pain, functional limitation, and discomfort when complicated by inflammation of the exostotic bursa, fracture, nerve compression, or malignancy^4,5^.

Although osteochondromas may develop in any bone undergoing enchondral ossification, they are frequently detected in the metaphysis of long bones, particularly the proximal humerus, proximal tibia, and distal femur. The involvement of small bones in the hands and feet is infrequent^5^. Solitary osteochondromas rarely occur in the foot and most often affect the metatarsals. Isolated tarsal osteochondromas are extremely rare, accounting for less than 1% of cases^2^.

We present a rare case of solitary osteochondroma of the medial cuneiform in a 22-year-old female who presented to us with a painful lesion on her foot that affected her daily activities and required surgical excision. The case has been reported for its rarity and educational value, emphasizing the need to consider surgical intervention when necessary. This case report has been reported in line with Surgical CAse REport (SCARE) 2023 Criteria^6^.

### Patient information

Our patient is a 22-year-old lady who presented to the outpatient department of our tertiary care hospital with a 2-year history of swelling on the dorsal aspect of her left foot, the size of which was gradually increasing. The swelling was initially painless but had been associated with pain for the past 2 months. She also complained of difficulty wearing footwear, especially while being involved in sports activities. The patient denied any traumatic events or sports injury. Moreover, night pains were not reported. The patient’s medical and surgical history is unremarkable. She reports no prior medication use and has no known allergies. A family history assessment revealed no similar illnesses. She also denies any history of smoking, alcohol consumption, or recreational drug use.

### Clinical findings

**Figure 1:**
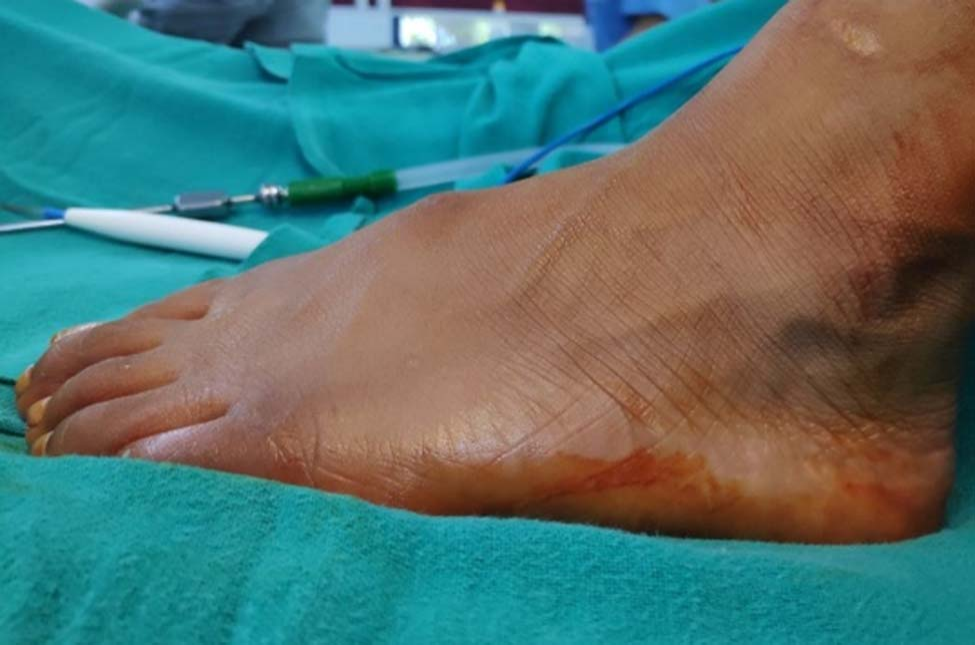
Clinical picture showing swelling over the dorsum of the left foot.

Clinical examination revealed approximately 2×2 cm swelling and tenderness at the medial aspect of the left foot along the first ray (Fig. 1). On palpation, a single hard, solid mass was noted. There were no dermatological, vascular, or neurological abnormalities. No other prominences were found in the skeleton.

### Diagnostic assessment and interpretation

**Figure 2:**
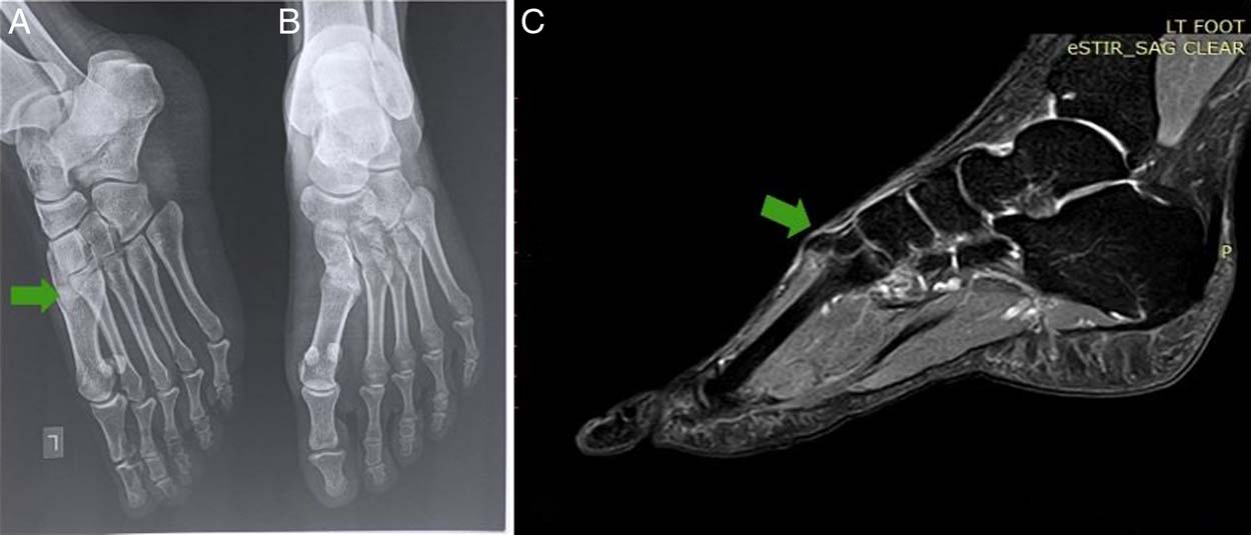
Radiograph of the left foot in oblique view (A) and anterior–posterior view (B) showing a radio-opaque lesion arising from the medial cuneiform. STIR MR sagittal section of left foot (C) showing a well-defined T1/T2 hyperintense and STIR hypointense bony outgrowth arising from the left medial cuneiform, with a thin cartilaginous cap.

Plain radiographs of the foot, oblique (Fig. 2A), and anterior–posterior (Fig. 2B) views showed a dorsal boss of the distal medial cuneiform. On further imaging, an MRI of the left foot showed triangular-shaped bony projection from the superomedial aspect of the dorsal medial cuneiform (Fig. 2C).

### Intervention

A course of conservative therapy in the form of analgesics, rest, and footwear modification was tried, which only provided temporary relief. As conservative therapy failed to alleviate the symptoms permanently, surgical treatment was warranted. The patient and her family were counseled, and surgical excision of the lesion was planned. The surgery was performed by a senior orthopedic surgeon and an orthopedic resident, with assistance from anesthesiologists, nurses, and intern doctors.

**Figure 3:**
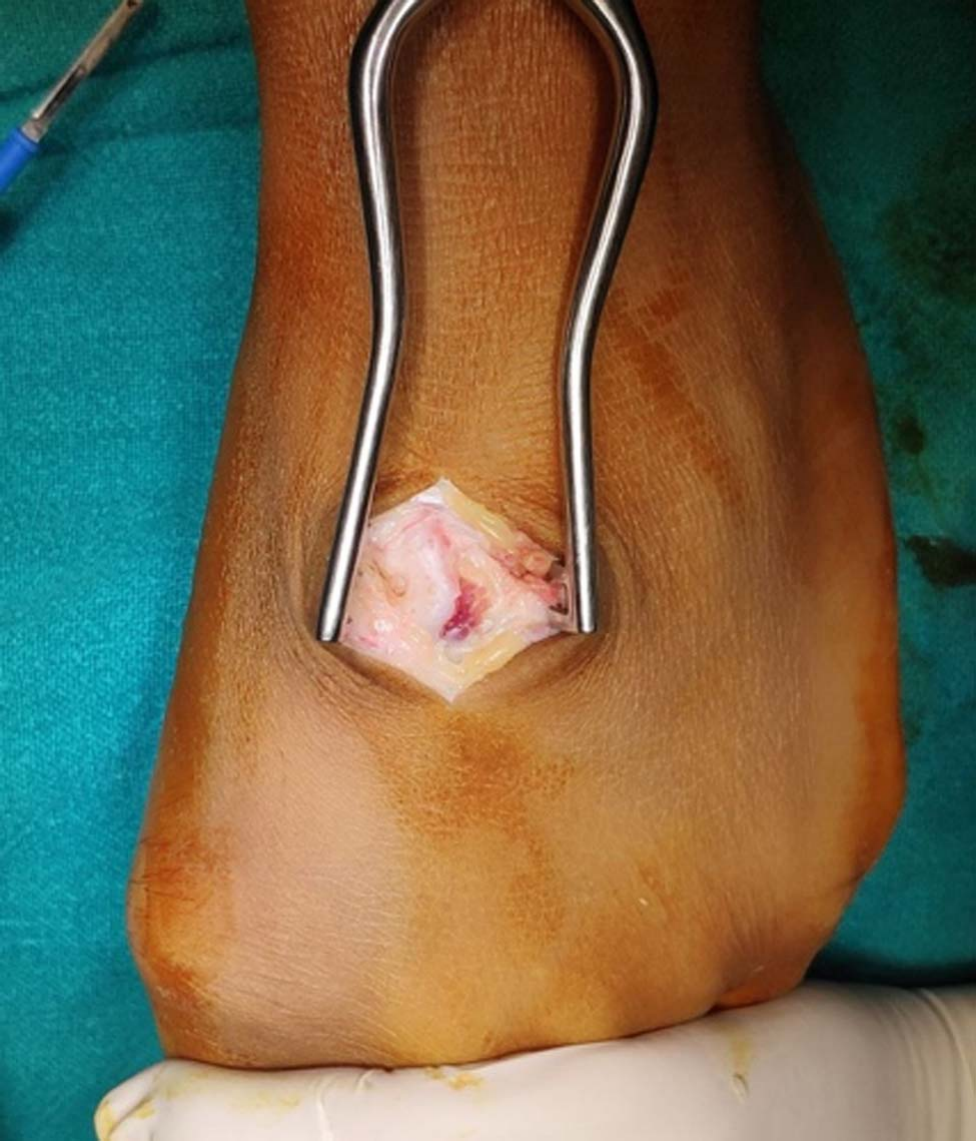
Intraoperative image of osteochondroma localized at the medial cuneiform.

During the operation, the patient was placed supine on the operating table with a side post for the leg and a foot post after adequate spinal anesthesia. The tourniquet was inflated, and a linear incision was made over the dorsum of the left foot. Subcutaneous tissues were dissected, tendons were retracted, and the entire tumor was exposed (Fig. 3). The tumor was then excised from the base. The wound was then closed in layers. The excised specimen, measuring 1 cm×0.5 cm, was sent for histopathological examination.

**Figure 4:**
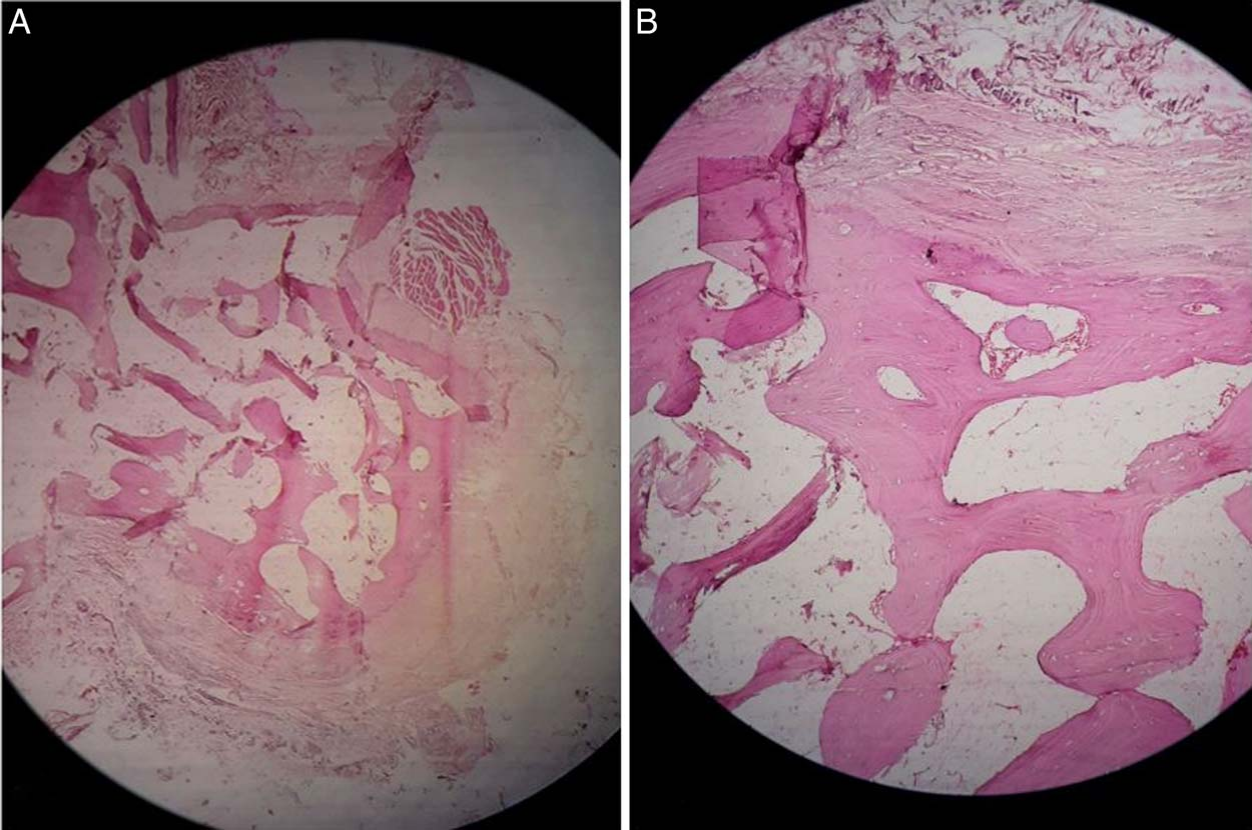
The histopathological section displays fragmented vitalized bony tissue with lacunae containing osteocytes. Additionally, the fibrocartilaginous stroma shows mild chronic inflammatory cell infiltration, congested thick and thin-walled blood vessels, and bundles of skeletal muscle fibers. There is no evidence of atypia. (A) ×4 magnification. (B) ×10 magnification.

The histopathology report showed predominantly cartilaginous tissues with no evidence of malignancy in the study specimen, leading to the conclusion that this is, in fact, a benign osteochondroma (Fig. 4).

### Follow-up and outcomes

Our patient was given standard postoperative care and had an uneventful recovery. Sutures were removed after 2 weeks, and gradual weight bearing was allowed as tolerated. The initial type of pain that she was experiencing was completely relieved, and she could wear her shoes without any difficulties. In 2 weeks, she had resumed her daily activities. Physical therapy to maintain ankle range of motion began 2 weeks postsurgery and continued for 2 months, with session duration and intensity adjusted based on recommendations from a physiotherapist. She was closely followed up in outpatient settings for a year. At her final check-up, she was pain-free and engaged in her daily activities as well as recreational sports. The scar was healthy and nontender, and there are no features suggestive of a recurrence of the tumor.

## Discussion

While they are a common entity, isolated osteochondromas of the foot are an extremely rare finding. The most common site for osteochondromas is the knee, which presents as painless bony protuberances around the growing ends of the tibia and femur^7,8^. When present in the foot, osteochondromas are commonly encountered in the metatarsals, with only a few cases affecting the cuneiform being reported to date^9–12^. To the best of our knowledge, our case is the third reported worldwide and the first reported in Asia of a cuneiform osteochondroma^10–12^.

Solitary osteochondromas are typically asymptomatic. When patients present with symptoms necessitating radiologic evaluation, the diagnosis of osteochondroma typically occurs incidentally^13^. The most typical osteochondroma symptom is a nontender, painless deformity associated with the slowly expanding exophytic tumor^14^. Osseous deformity, fracture, vascular compromise, neurologic sequelae, overlaying bursa formation, and malignant transformation are other consequences that result in symptoms^15^. In our patient, the pain was most likely caused by the mass effect on the bursae and, consequently, nervous tissue overlying the superficial layers above the mass.

On radiography, osteochondromas are pathognomonic for the cortical and medullary bone that protrudes from and is continuous with the underlying bone^14^. With osteochondromas, the three-dimensional imaging capacity of computed tomography frequently enables the best representation of the pathognomonic cortical and marrow continuity of the lesion and parent bone^16^.

Individualized osteochondroma treatment involves follow-up and supportive care for small asymptomatic or minimally symptomatic lesions, with larger symptomatic lesions potentially requiring resection at the base where there is continuity to the underlying bone^14^. Small bone osteochondromas have an ill-defined margin, which increases the likelihood of recurrence following tumor excision^17^. In our patient, there has been no recurrence in 1 year; however, we will be following upon the case on a regular basis to check for signs and symptoms suggestive of recurrence. In this case, an excisional biopsy above the tumor base was done.

### Strengths and limitations

This case report highlights the efficacy of an excisional biopsy for osteochondroma in the cuneiform bone, as evidenced by a positive response and no recurrence during a 1-year follow-up. However, its single-case nature limits generalizability, and longer follow-up is needed to fully assess long-term outcomes.

## Conclusion

Osteochondroma in the foot is a rare occurrence. When present, they may cause pain and interfere with daily activities. While conservative therapy remains the mainstay of treatment for osteochondroma, once it is ineffective in providing symptomatic relief, surgical excision is advisable with evidence of a good outcome.

## Ethical approval

Ethical approval for this study is not required by our institution.

## Consent

Written informed consent was obtained from the patient for publication of this case report and accompanying images. A copy of the written consent form is available for review by the editor-in-chief of this journal upon request.

## Source of funding

Funding was not received for this study.

## Author contribution

R.S.: concept, manuscript preparation, edit and review, and guarantor; S.S.M.: manuscript preparation, data collection, obtaining consent from the patient, manuscript editing, and review; A.P.: manuscript preparation, editing, and review; K.P.: manuscript preparation, editing, and review; A.P.: manuscript preparation, editing, and review.

## Conflicts of interest disclosure

The authors declare no conflicts of interest.

## Research registration unique identifying number (UIN)

1. Name of the registry: not applicable.
2. Unique identifying number or registration ID: not applicable.
3. Hyperlink to your specific registration (must be publicly accessible and will be checked): not applicable.

## Guarantor

Rohit Shrestha.

## Data availability statement

Data sharing is not applicable to this article.

## Provenance and peer review

Not commissioned, externally peer-reviewed.
